# Advantages of individualizing the placenta accreta spectrum management

**DOI:** 10.3389/frph.2022.1096175

**Published:** 2023-01-06

**Authors:** José Miguel Palacios-Jaraquemada, Álbaro Jose Nieto-Calvache, Rozy Aditya Aryananda, Nicolás Basanta

**Affiliations:** ^1^ Department of Gynaecology, Otamendi Hospital, Buenos Aires, Argentina; ^2^ Clínica de Espectro de Acretismo Placentario, Fundación Valle del Lili, Cali, Colombia; ^3^ Latin American Group for the Study of Placenta Accreta Spectrum, Cali, Columbia; ^4^ Dr. Soetomo Academic General Hospital, Universitas Airlangga, Surabaya, Indonesia; ^5^ Department of Obstetrics an Gynaecology, Hospital General de Agudos Juan A Fernández, Buenos Aires, Argentina

**Keywords:** placenta accerta, individualize, PAS management, hysterectomie, topographic classification

Placenta accreta spectrum (PAS) is an entity with a wide range of clinical presentations. From cases with “mild” lesions requiring moderate surgical effort and are associated with few complications (1), to severe ones with a challenging management and life-threatening risk (2).

Individualizing the management of PAS patients is essential, especially when a significant percentage of women undergoing PAS surgery ultimately do not have this diagnosis or present superficial or focal lesions (3). In addition, let's not forget that some women wish to preserve their fertility (4) or that vascular interventions or the hysterectomy itself can cause additional morbidity (5).

However, customizing the management of PAS seems complicated. Choosing the ideal management for each patient is a task for personal and institutional reasons.

Although multiple treatments have been described (6, 7), frequently, each PAS team adopts one strategy for all cases and specializes in its performance. Applying this type of intervention becomes the norm for all patients with PAS presenting at a specific hospital. Additionally, historic international consensus focuses on hysterectomy as the standard treatment, mentioning other management alternatives as secondary options “only” for exceptional cases (6, 8). There is practically no doubt that supervised training modify this previous concept.

On the other hand, the preoperative classification based on the severity of the placenta invasion needs a subsequent histological analysis (9). Therefore, pathologic tissue analysis does not help make decisions on the table, and histological study is subject to multiple biases.

The placenta invasion topography is closely related to surgical complexity and maternal morbidity (10). Placenta invasion below the peritoneal reflection implies more significant risks due to reduced space, extrauterine arterial pedicles’ multiplicity, and requirement for adequate vascular control strategies (11, 12).

It is essential to validate an applicable classification before non-reversible maneuvres (that is, before incising the uterus and causing bleeding) that also suggests a specific type of treatment according to the characteristics of each case. The accurate dissection of coalescence pelvic fascia's spaces allows using avascular spaces with minimal tissue manipulation to have a precise diagnosis and avoid possible complications, this is the principle of intraoperative PAS staging. Additionally, the surgical staging allows getting objective PAS information that could be missing after prenatal ultrasound (13).

Although it is possible to make a diagnostic approach based on the results of prenatal images (ultrasonography and MRI), it is during the laparotomy for the cesarean section when the surgeon can use safe and straightforward dissection techniques to establish the affected area of the uterus, and consequently, the risk of massive bleeding or organ injury.

Topographic classification seeks to define which uterine wall is affected (anterior, lateral, or posterior), the presence of lesions above the level of peritoneal reflection (high lesions) or below that level (low or “subperitoneal” lesions), and the nature of the lesion (characterized by neovascularization or with the presence of fibrosis between the uterus and neighboring organs). The main objective of the intraoperative topographic classification of PAS (14, 15) is to use the most suitable PAS treatment according to objective surgical findings.

**Figure 1:**
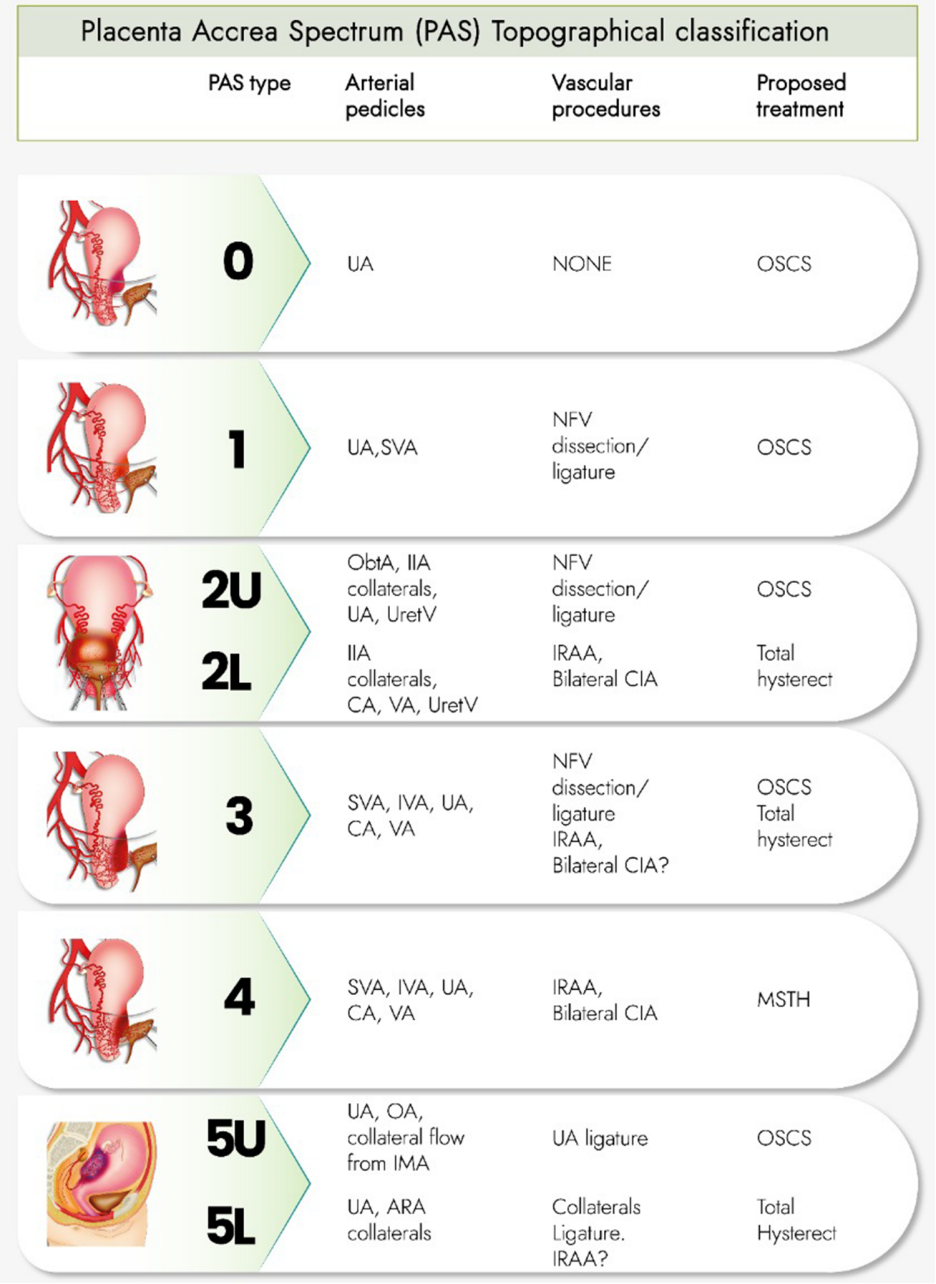
Intraoperative staging makes it possible to determine which uterine wall is affected and the relationship of the lesion to the vesicouterine peritoneal fold (above or below that level), as well as the predominance of neovascularization or the presence of vesicouterine fibrosis.

Each of the possible affected uterine areas is related to well-defined anatomical arterial pedicles and neighboring structures (urinary and vascular) that determine the surgical difficulty and the recommended dissection and management strategies (16) (Figure 1).

The topographic classification establishes a “conceptual 3D map” and different management options for each case. For example, what the PAS teams call “A, B, and C plans,” necessary when facing the wide variety of PAS clinical presentations, does not necessarily require the same management route in all cases.

Some publications described the advantages of topographic classification in retrospective studies (14–16). Hopefully, the comparison between individualized arterial pedicles control and the mandatory use of interventional radiology in all the cases could be promissory in large prospective multicentric studies. Likewise, the topographic classification, followed by a protocolized and individualized management, can enhance the postoperative histological analysis.

In each topography, some arterial pedicles are identified that provide most of the irrigation to the PAS area and that determine the recommended vascular procedures and the type of treatment necessary (One Step Conservative Surgery [OSCS], Total hysterectomy or Modified SubTotal Hysterectomy [MSTH]).

Type 0 PAS: uterine “window” or dehiscense. Type 1 PAS: uterine segment upper part involvement. Type 2 PAS: parametrial involvement (2U: upper parametrial involvement, 2 L: lower parametrial involvement). Type 3 PAS: cervix or uterine segment lower part involvement (below the peritoneal reflection). Type 4 PAS: type 3 PAS plus vesicouterine fibrosis. Type 5 PAS: uterine posterior wall involvement (5U: involvement of the upper part of that wall. 5 L: Lesions below the level of the peritoneal reflection).
